# Does the Weekly Practice of Recalling and Elaborating Episodes Raise Well-Being in University Students?

**DOI:** 10.1007/s10902-022-00547-w

**Published:** 2022-07-06

**Authors:** Angelica Moè

**Affiliations:** Department of General Psychology, University of Padua, Padua, Italy

**Keywords:** Well-being, Need satisfaction, Need frustration, Self-compassion, Emotion regulation, Gratitude, Self-affirmation, Goal setting

## Abstract

Increasing well-being is a prominent worldwide goal that can be achieved primarily through social support and environmental factors. However, in times of social distancing or isolation, it is important to also rely on self-managed activities. This study aimed to (a) test the effectiveness of a seven-week well-being intervention, in increasing need satisfaction, self-compassion, emotion regulation, and grateful disposition by curbing need frustration, self-derogation, and emotional suppression, and (b) examine the maintenance and long-term effects of the practices based on recall, elaboration, and writing. One hundred and twenty university students weekly recalled and elaborated for seven consecutive weeks on three recent episodes of gratitude, self-affirmation, goal setting, or meaningful things, according to the group to which they were assigned. Before the intervention, immediately after and one month later, they filled in questionnaires to assess need satisfaction/frustration, self-compassion/derogation, emotion regulation and grateful disposition. The results confirmed an increase in well-being and a decrease in ill-being for all groups (Cohen *d* for the significant differences ranging from 0.18 to 0.53). The effects were maintained one month later and even increased for self-compassion, self-derogation, need frustration, and emotional reappraisal. A follow-up assessment revealed that a third of the participants continued with the well-being practices. Implications and suggestions for future well-being interventions are discussed.

## Introduction

Rising well-being is a growing worldwide priority, mostly in these times characterized by uncertainty due to the pandemic outspread (e.g., Ludovic, et al., 2020; McBride et al., 2021). The last World Happiness Report (Helliwell et al., 2021) lists both protective and risk factors that could favor or impair well-being during the COVID-19 pandemic. Among protective factors, one of the most prominent is the feeling of psychological connection despite the recommended physical distancing.

How can we reach this experience of connectedness while reducing personal contacts with people, maybe due to working or schooling at home? A solution could be to nurture the *perceived* satisfaction of the basic psychological needs as theorized by Self-Determination Theory (SDT: Deci & Ryan, 2017): Human beings can express their full potential (and experience well-being: Martela & Sheldon, 2019) as much as they feel satisfied rather than frustrated the three interrelated needs for relatedness/acceptance (feeling connected and supported), competence (experiencing to master tasks successfully) and autonomy (feeling a sense of direction, meaning, and choice).

Need satisfaction leads people to express high levels of well-being and also to face successfully life challenges (e.g., Tang et al., 2020; Walker & Kono, 2018). Instead, need frustration leads to ill-being and maladaptive behaviors, thus resulting in reduced well-being (Church et al., 2013) and motivation (Moè & Katz 2020, 2021). Therefore, how can we improve satisfaction and reduce the frustration of basic psychological needs?

A bulk of research (for reviews, see Ryan & Deci, 2020; Ntoumanis et al., 2020) demonstrated that need satisfaction is favored by an autonomy supportive social environment, characterized by expressing acceptance, showing an understanding of the other perspective, providing meaningful choices, encouraging, and supporting efforts toward success (see, e.g., Reeve, 2009, 2016).

Only rarely have personal resources and self-managed practices been considered as a way to increase the satisfaction of the needs. For example, Behzadnia and FatahModares (2020) involved participants in a series of 10-day exercises grounded in SDT and confirmed that they were successful in increasing need satisfaction and reducing need frustration. In fact, what makes the difference is not the objective need satisfaction or frustration, but the *perceived* satisfaction or frustration. From a practical point of view, this is an important issue, because during the isolation and physical distancing imposed to reduce the spread of COVID-19, there are more opportunities to engage in self-managed well-being practices than in need supportive social contexts.

### The Well-Being Practices

Over the years, many well-being practices have been proposed to be enacted individually, based on anchoring techniques, that is, on sensing, remembering, and elaborating on meaningful episodes, such as satisfying relationships, achieved goals, and personal strengths. Anchoring with past successful and mastery experiences (that is, replicating/remembering positive sensations and images) rises well-being, as well as adopting the technique AIM, developed by Diener and Biswas-Diener (2011), which implies the three steps of paying Attention (to positive episodes), giving a positive Interpretation (as satisfactory, successful, self-realizing), and Memorizing them (which means linking with the Self-representations and building a satisfactory view of ourselves).

Both anchoring and AIM techniques base their effectiveness on increased positive affect through upward spirals, following the broaden-and-build theory (Fredrickson & Branigan, 2005; Fredrickson & Joiner, 2002) and increased self-determination (Ryan & Deci, 2017), which previous research has shown to relate to well-being (e.g., Deci, & Ryan, 2008; Milyavskaya & Koestner, 2011) also during the Covid-19 outbreak (e.g., Šakan et al., 2020).

Among the anchoring/recalling techniques proposed, given the objective to raise need satisfaction and curb need frustration, this study considered the following: gratitude list (Emmons & McCullough, 2003), self-affirmation (Fein & Spencer, 1997), goal setting (Locke & Latham, 1990) and three good things in life (Seligman et al., 2005).

The gratitude list requires people to think about the situations in which they experienced gratitude and to report them by elaborating and savoring these experiences. It was first developed by Emmons and McCullough (2003), and then successfully applied to a range of populations, such as college students (Işık & Ergüner-Tekinalp, 2017), adolescents (Froh et al., 2008), teachers (Chan, 2010), the elderly (Killen & Macaskill, 2015) and prisoners (Deng et al., 2018). Although the results overall demonstrated an increase in well-being indicators and a decrease in ill-being, independently of the gratitude target (Berger et al., 2019), this was not always the case as outlined by Davis et al. (2016) and Dickens (2017), who suggest the need to consider some moderating factors, such as positive affect (see, e.g., Froh et al., 2009; Wood et al., 2010), and the practices in which the control group is involved.

Self-affirmation was first proposed by Fein and Spencer (1997). It consists in making salient personal strengths or values, fields, and activities in which we typically succeed so that to increase the perception of competence and self-worth. It can be applied spontaneously (Emanuel et al., 2018), and its effectiveness in increasing well-being has been demonstrated, for instance, by Armitage (2016) and Nelson et al. (2014), mainly for those participants low in baseline well-being (for a review, see Cohen & Sherman, 2014).

Goal setting was developed by Locke and Latham (1990) and consists in setting relevant and achievable goals. Successful completion of personal goals should enhance need satisfaction, thus favoring well-being. A confirmation of this rationale comes from studies demonstrating that goal setting is related to well-being (see, e.g., Grégoire et al., 2012; Dulagil et al., 2016).

Thinking about meaningful things has been included as an active control condition, involved in doing something the experimenter can control. It is quite similar to the technique ‘three good things in life’ proposed by Seligman et al. (2005), but the possibility of choosing to report negative meaningful episodes. Hence, depending on the content of the episodes reported, it is possible to predict either that this kind of recalling and elaborating would increase well-being or that it would not.

Considering the main goal of this study, the effectiveness of well-being practices based on the implementation of strengths through weekly exercises will be evaluated first on need satisfaction and frustration, predicting, respectively, an increase and a decrease for all groups. The gratitude list practice should mainly favor satisfaction and reduce frustration of the need for relatedness, since it refers to the experience of having received help from others. Self-affirmation practice should primarily increase satisfaction of the need for autonomy and reduce frustration of the same need, as it refers to the experience of self-management of personal strengths. Goal setting is expected to increase satisfaction and reduce frustration of the need for competence, since it refers to the fulfillment of personally endorsed goals. Since the three needs are interrelated, changes in overall need satisfaction and frustration are expected.

Then, other well-being aspects linked with need satisfaction/frustration are expected to increase and ill-being aspects to decrease. Previous research (Moè & Katz, 2020, 2021) found that need satisfaction is related to self-compassion (a kind, not judgmental attitude toward oneself: Neff, 2003a), and emotional reappraisal (an effective emotion regulation strategy: Gross, 2015), while need frustration is linked with self-derogation (a judgmental attitude, expression of poor self-compassion) and emotional suppression (an ineffective emotion regulation strategy). Therefore, it is predicted that these well-being practices will also increase self-compassion and emotional reappraisal and curb self-derogation and emotional suppression. Finally, a grateful disposition as an expression of well-being and openness (McCullough et al., 2002) is also expected to increase.

### Study Aims and Hypothesis

This study will address for the first time the effectiveness of online implemented well-being practices based on recalling and elaborating episodes of gratitude, self-affirmation, or goal setting during the Covid-19 outbreak. The first goal is to demonstrate that these practices will increase well-being and decrease ill-being. Specifically, an increase is predicted from the beginning to the end of the 7-week practice in need satisfaction, self-compassion, emotional reappraisal, and grateful disposition, and a decrease in need frustration, self-derogation, and emotional suppression. The second goal is to verify that the effects maintain over time and that participants are likely to continue with the practices.

The following hypotheses lead to the research.

#### Hypothesis 1a

Need satisfaction, self-compassion, grateful disposition, and reappraisal increase from the beginning to the end of the well-being practice period;

#### Hypothesis 1b

Need frustration, self-derogation, and suppression decrease;

#### Hypothesis 2

The effects maintain: In the follow-up assessment the scores are the same or higher than right after the end of the intervention;

#### Hypothesis 3

The participants are satisfied with the well-being practices and self-report to continue with them in the follow-up assessment.

## Method

### Participants and Procedure

The study was approved by the local Ethics Committee (University of Padua, Psychological area, protocol n. 3772). Written consent was obtained from each participant for participation. A large convenience group of Italian university students attending bachelor or master degrees in psychology were proposed to participate in an online study lasting 7 weeks (from October 8 to November 20, 2020) in return of 1-point course credit. One hundred ninety-nine agreed and filled in the online questionnaires (see Measures) at t0 (before the beginning). Then, for 7 consecutive weeks, they were invited to individually perform one of the well-being practices based on recalling three episodes of gratitude, self-affirmation, goal setting or meaningful episodes, depending on the group to which they were assigned, to write them in an online box, and to elaborate on them; see Table 1 for details. Anonymity was a guarantee so that they could freely report the episodes they remembered and elaborated on: they were not asked for their names, but solely for gender and age. The pairing was made using a personal code.

**Table 1:** Messages given weekly to participants

To everybody	
Choose a place where you can stay quiet and undisturbed for approximately 15–20 min. Set a day and time and keep it as long as possible every subsequent week, for example, Sundays at 6 pm in your bedroom. Now, think about the last week, then	
Condition	
Gratitude list	Write three episodes in which you experienced gratitude to someone (for example, you received a nice phone call). Please be specific: what happened, which relations you have with those persons
Self-affirmation	Write three episodes in which you displayed personal strengths. For example, you value courage and felt that you are courageous. Write down a description: what happened, who was with you
Goal setting	Write three goals that you achieved (for example, you were successful in finishing a book). Describe the goals, how important they are to you, what you did and happened
Thinking about meaningful things	Write down three meaningful episodes. Describe what happened, who was with you, what they did, and how important these things are to you
To everyone (to be done individually, anonymity guarantee)	
Describe your expectations, what happened next, the meaning you gave to the episodes, how you felt	
Now, sit down, relax, and stay 3 min to review one that of these episodes, the one you like the most. You could close your eyes and let the scenes run as in a video	

One hundred and eighty-nine continued by filling out the questionnaires in t8 and performing the well-being practices. After one month (December 21^st^), they were contacted again to fill in the same instruments a last time and asked if they were still doing the exercises and their level of satisfaction (see the Appendix). The pandemic outspread at that time was growing. The lessons were delivered in the room and also online. Students could choose to attend in presence or not. Due to mobility restrictions, no vaccine yet, and the fear of getting infected, the large majority of the students preferred/were forced to attend lessons online, frequently also being in lockdown.

The final sample consisted of 120 students, Mage = 22.99, SD = 4.53, randomly assigned to the following groups: gratitude list (n = 32, 3 males), self-affirmation (n = 31, 3 males), goal setting (n = 32, 5 males), meaningful things (n = 25, 4 males), who performed the practices 5 (n = 3), 6 (n = 20) or 7 (n = 97) times. To confirm that those who missed the follow-up session (n = 69) did not differ from those who participated (n = 120), a series of Student’s t tests were performed that compared mean scores in t8 of the two subgroups. None of the differences were significant, suggesting that there was no self-selection bias.

A post hoc power analysis with G*Power (Faul et al., 2007) showed that the critical *F* is 2.68, to detect an effect of *f* = 0.25, *p* < 0.05 with 120 participants for a between factors repeated measures ANOVA 4 (groups) × 3 (times).

## Measures

The following questionnaires were included in the online survey and presented in the order they are described to all the groups at t0, t8, and follow-up.

*Basic Psychological Need Satisfaction and Frustration scale* (BPNSNF: Chen et al., 2015), in the Italian validation by Costa et al. (2018). It presents 24 items assessing perceived need satisfaction (example item ‘I feel that my decisions reflect what I really want’), or frustration (example item ‘I feel pressured to do too many things’) to be rated on a 5-point Likert scale (1 = completely disagree to 5 = completely agree). Two scores were computed by averaging the 12 items referring to each dimension.

*Self-Compassion Scale* (Neff, 2003b), in the Italian validation by Voci et al. (2019). It consists of 26 items, 13 of which assess self-compassion (example item ‘When I’m going through a very hard time, I give myself the caring and tenderness I need’), and 13 self-derogation (example item ‘I’m disapproving and judgmental about my own flaws and inadequacies’) to be rated on a 5-point-Likert scale ranging from 1 = almost never to 5 = almost always. Two scores were computed by averaging the 13 items that refer to each scale.

*Emotion Regulation Questionnaire* (Gross & John, 2003), in the Italian validation by Balzarotti et al. (2010). It consists of 10 items that assess reappraisal (example item ‘I control my emotions by changing the way I think about the situation I’) or suppression (example item ‘I control my emotions by not expressing them’) to be rated on a 7-point Likert scale (1 = totally disagree to 7 = completely agree). Two scores were calculated by averaging the six reappraisal items and four suppression items.

*Gratitude Questionnaire* (McCullough et al., 2002), Italian validation by Caputo (2016). It presents six items (example ‘I have so much in life to be thankful for’) to be rated on a 7-point Likert-type scale (1 = strongly disagree to 7 = strongly agree). An average score was obtained after having reversed items 3 and 7.

The Cronbach alphas for each measure at t0 are reported in Table 2.

**Table 2:** Mean scores and SD (in brackets) for the assessed variables 3 days before the beginning (t0), 3 days after (t8) and 1 month after (follow-up) the well-being practice period, together with the statistical values for the main effect of time

Variable	Cronbach α	t0	t8	Follow-up	*F*(1, 116)	*p*	_*p*_*η*^2^	*Post-hoc p* < *.05*	*Cohen d t0-t8*
Need satisfaction	0.87	3.75 (0.58)	3.90 (0.56)	3.88 (0.52)	12.80	0.001	0.10	t0 < t8 = FU	0.30
Need frustration	0.85	2.08 (0.65)	2.00 (0.62)	1.95 (0.62)	9.01	0.003	0.07	t0 > FU	0.14
Self-compassion	0.86	2.85 (0.68)	3.01 (0.68)	3.12 (0.71)	30.01	0.001	0.21	t0 < t8 < FU	0.27
Self-derogation	0.87	3.39 (0.74)	3.03 (0.72)	2.92 (0.69)	85.26	0.001	0.42	t0 > t8 > FU	0.53
Reappraisal	0.89	4.60 (1.23)	4.72 (1.21)	4.87 (1.19)	8.77	0.004	0.07	t0 = t8 < FU	0.011
Suppression	0.75	3.47 (1.33)	3.24 (1.46)	3.18 (1.41)	11.16	0.001	0.09	t0 > t8 = FU	0.18
Grateful disposition	0.82	4.71 (0.99)	4.86 (0.91)	4.87 (0.95)	7.30	0.008	0.06	t0 < t8 = FU	0.18

### Data Analysis

To test H1 and H2, a series of ANOVAs of 4 (groups) × 3 (times) was performed on each of the variables evaluated. Then Student t-tests were run as a post-hoc analysis. To test H3 frequencies analyses were run. Finally, to better understand the underlying mechanisms, a content analysis was run.

## Analyses and Results

### Preliminary Analyses

**Fig. 1:**
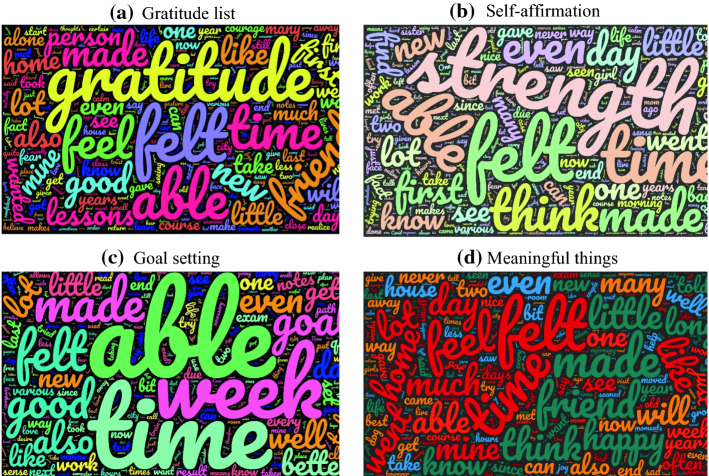
Content of the episodes reported by the four groups

Examples of reported episodes are ‘I unexpectedly met a friend I have never seen for years: we decided to have lunch together’, ‘I went to visit my grandmother and played with my nephew. It was so lovely and we had a great time’ (gratitude list), ‘I was able to involve my brother in doing housework. I felt happy and satisfied’, ‘In a volleyball match I got a point: I felt satisfied and proud for my efforts’ (self-affirmation), ‘I succeed in studying all the chapters and passing the exam’, ‘I succeed in watching the football match with my family’ (goal setting) and ‘I got on the bus and found out that I was without a ticket. I felt so anxious and I was waiting for the next bus stop’, ‘I spent a lot of time with my best friend; I know I can trust her for everything’ (meaningful things). To visualize what they wrote, 4 word-clouds were created after having translated all the episodes reported by each group. As can be seen in Fig. 1, all groups used the words ‘feel’ or ‘felt’, ‘made’, ‘able’ and ‘time’, confirming that they reported feelings and/or things done during the week. However, they differed in the kind of words reported more frequently. In fact, the group gratitude list most frequently reported the words ‘gratitude’, the group self-affirmation ‘strength’ and the goal setting ‘week’. This clearly shows that they focused on grateful episodes or on their strength or goals achieved in a timely manner (week). The meaningful things group varied a lot in the kind of words used, such as ‘home’, ‘friend’, ‘happy’, ‘like’. None of these words was as frequent as ‘gratitude’, ‘strength’ and ‘week’ in the other three groups. This confirms that the four groups engaged in the task required, as they reported episodes consistent with the instructions given.

Finally, the proportion of positive/negative/neutral episodes reported by the meaningful things group was calculated, following the procedure described by Emmons and McCullough (2003), page 379. Specifically, an assistant researcher coded the episodes as positive if pleasant, negative if unpleasant, and neutral if neither pleasant nor unpleasant. Typically, positive episodes referred to success, good relationships or achievements (examples ‘I really started lessons and I like the topics: I am happy for the choice of this master degree, ‘I celebrated my birthday with the whole family’, ‘I spent an entire afternoon talking to my father after weeks I have not seen him’), while negative events were about failures, disappointments, and refusals (examples ‘The professor told us that we have to study two books instead of one: it is a great deal of material’, ‘My boyfriend failed an exam and cannot graduate this month’, ‘I quarreled with my aunt’). The few doubts were resolved by discussion. In this way, the episodes were coded as follows: positive 67%), negative (32%), and neutral (1%). This emphasizes that the participants in the meaningful things group focused on the most significant things that occurred during the past week, not only on positive and successful episodes as done by the first three groups.

### Effectiveness of the Well-being Practices

The main effect of time was significant for all the variables assessed; see Table 2 for means, statistical values, and effect sizes. The post-hoc analyses showed that there was a significant increase from t0 to t8 in need satisfaction, self-compassion, and grateful disposition and a significant decrease in self-derogation and emotional suppression. The effects sizes ranged from small to medium in size, ranging from.18 to.53 (see Table 2).

None of the interactions were significant: well-being (need satisfaction, self-compassion, and grateful disposition) increased and ill-being (self-derogation and emotional suppression) decreased for all groups, suggesting that recalling, elaborating, and writing weekly episodes is beneficial irrespective of the content. However, need frustration and emotional reappraisal did not change from t0 to t8.

### Maintenance of Effects

The Student’s t-test comparisons between scores at t8 and at one month of follow-up showed that for need satisfaction, emotional suppression and grateful disposition effects were maintained, while for need frustration and self-derogation there was a significant decrease and for self-compassion and emotional reappraisal a significant increase. These results showed that well-being increased and ill-being decreased in 4 of 7 aspects one month after the end of the 7-week well-being practices.

### Satisfaction and Willingness to Continue

Approximately one participant out of three declared to continue with the well-being practice: 15% regularly and 18% sometimes. Furthermore, 40% reported that they had thought about the experience and suggested others to adopt the well-being practices. The mean level of satisfaction was 3.03, SD = 0.92, see the Appendix for details on the percentages.

### Content Analysis

The episodes written during the 7 weeks were scored with the help of a research assistant. Based on the assumptions of SDT, they were coded as referring to (a) warmth and satisfying relationships, for example, with friends, family and mates; (b) personal achievements and related feelings of competence; or (c) successful choices/expressions of autonomy or dominance, which refer, respectively, to the satisfaction of the needs for relationships, competence, and autonomy.

Table 3 reports the mean number of written episodes for each category, overall and by the four groups. The post-hoc comparisons through Student t-tests showed that most of the episodes were about relationships and achievements, suggesting that remembering positive relationships and successful experiences lead to the increase in well-being and decrease in ill-being.

**Table 3:** Mean number and SD (in brackets) of episodes written by the four groups

	Relationships	Competence	Autonomy
Gratitude list	14.01 (5.86)^a^	1.34 (1.49)^b^	0.44 (0.88)^c^
Self-affirmation	3.93 (3.85)^b^	6.68 (3.34)^a^	4.43 (2.83)^b^
Goal setting	3.44 (3.49)^b^	10.96 (6.25)^a^	1.97 (2.63)^b^
Meaningful things	9.72 (4.37)^a^	3.88 (2.71)^b^	3.60 (2.89)^b^
Overall	7.69 (6.30)^a^	5.82 (5.34)^a^	2.53 (2.85)^b^

*Note* The means that do not share a subscript differ significantly at *p* < 0.05

Considering the four groups separately, the gratitude list and the meaningful things group reported above all episodes about relationships, the self-affirmation and goal setting groups episodes in which they experienced competence. For the gratitude list group, this is in line with the instructions given, based on remembering people to be grateful for. For the meaningful things group, this result suggests that people tend to prefer to remember and focus on relationships more than on achievements or personal choices. The self-affirmation and goal setting groups focused more on achievements and successful experiences than on relationships or autonomy. For the self-affirmation group, this is less in line with what was predicted. In fact, the mean number of autonomy episodes is the largest among the four groups, but it is lower than the number of episodes referring to achievements and perception of competence.

## Discussion

This study tested for the first time the effectiveness and maintenance of self-managed well-being practices based on recalling and elaborating episodes of gratitude, self-affirmation, goal setting, or meaningful things during the COVID-19 outbreak. The results confirmed that these practices individually implemented by the participants increased need satisfaction, self-compassion, emotional reappraisal, a grateful disposition, and reduced need frustration, self-derogation, and emotional suppression.

**Fig. 2:**
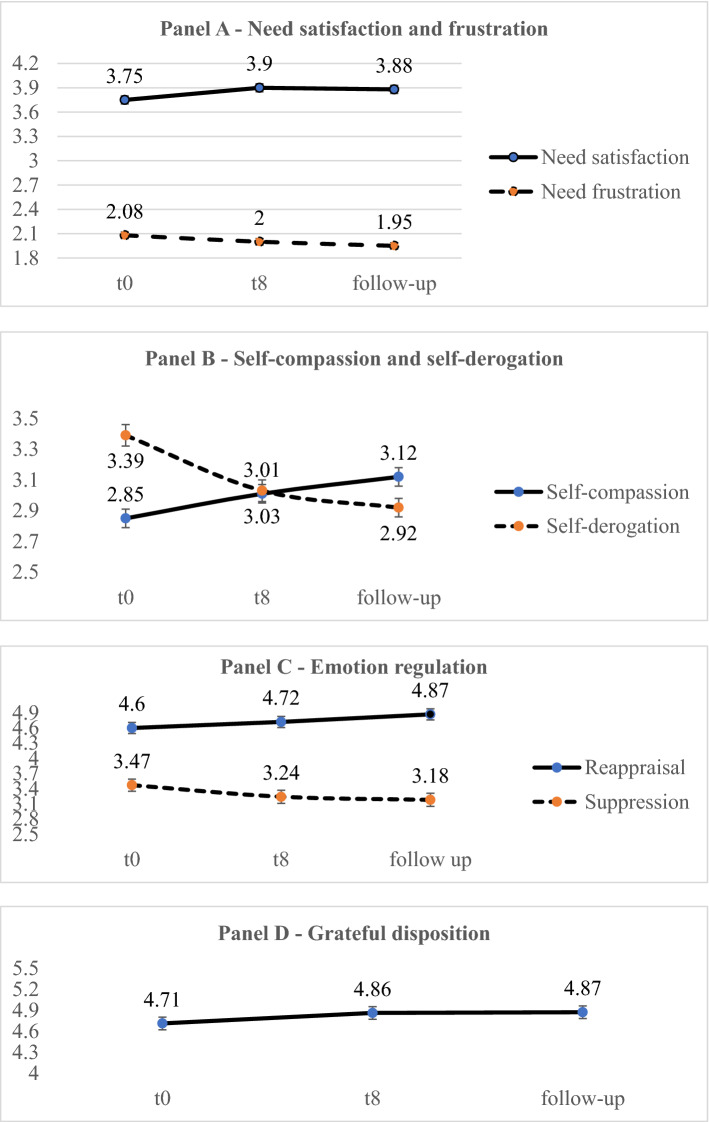
Increase in well-being and decrease in ill-being from t0 to t8 and in follow-up one month after the period of well-being practices. The error bars refer to SE

The effects were maintained one month after the end of the 7 weeks. Furthermore, self-compassion and emotional reappraisal even increased, need frustration, and self-derogation decreased, suggesting large maintenance effects; see also Fig. 2. As shown, the decrease in ill-being was greater than the increase in well-being, at least for self-derogation compared to self-compassion and suppression compared to reappraisal. Only for need satisfaction, the increase was greater than the decrease in need frustration (see also Table 2 for effect sizes). This effect could be linked to the baseline mean value, which was particularly low for need frustration suggesting little room for further decrease.

Interestingly, one out of three participants reported that (s)he was still doing the well-being practices one month after the end of the 7 weeks, and 40% suggested them to at least one person, thus confirming that they were appreciating the benefits.

### Increasing Need Satisfaction and Well-Being through Self-Managed Practices

A supportive social environment favors need satisfaction and well-being (Ryan & Deci, 2017, 2020). However, from a practical point of view, it is not always possible to rely on such a social environment (maybe because you work in a harsh context or your teacher or parents are controlling) or on any environment (because you are in quarantine or must respect social distancing rules). In these situations, you can apply personally self-managed well-being activities aimed at recalling and elaborating episodes of gratitude, self-fulfillment, and achieved goals. The results of this study confirm that they are effective, as they increase need satisfaction and reduce need frustration, as found by Behzadnia and FatahModares (2020) with 10 daily sessions with varying activities. Furthermore, this research showed that the effects extended to self-compassion, emotion regulation, and a grateful disposition. Finally, they maintained or even increased after one month. This is a very important result. The well-being practices were still producing their effects or even larger effects one month later. Notably, the participants who completed the questionnaires in the follow-up session were not those more motivated or experiencing higher levels of well-being: their scores did not differ from those who skipped the follow-up session. This enforces the result that the effects are long lasting. In fact, the last questions about ‘still practicing’ revealed that 1 participant out of 3 appreciated the practices so much to continue with them one month after the end of the planned period.

Notably, the most significant effects were about decrease in ill-being. As shown in Table 2 and Fig. 2, self-derogation decreased more than self-compassion increased with an effect size of up to 0.50, which means a medium effect. Similarly, the less functional emotion regulation strategy of suppression decreased more than the most effective of reappraisal increased. This effect could be explained as a ‘bad is stronger than good’ effect (Baumeister et al., 2001). Typically, people pay more attention to threats, losses, or generally ‘bad’ things they want to avoid and skip, thus devoting less attention to satisfying and good events. The practice of recalling and elaborating on positive events of gratitude, achieved goals, and personal strengths could have contrasted with this tendency, thus resulting in a larger decrease in ill-being, as well as a moderate, but nevertheless significant increase in well-being.

Self-compassion is a very important expression of self-acceptance and a source of well-being (Neff, 2003a). The results achieved in this study show that it increased greatly being the variable showing the largest effect sizes. In addition to well-known and tested trainings to increase self-compassion (for a review see Barnard & Curry, 2011), the results here obtained show that self-managed practices can be effective and maybe complement the structured trainings or be an alternative when restrictions due to the pandemic outspread limit the possibility to engage in group activities.

Improving emotion regulation skills can reduce the occurrence of psychopathology and increase happiness (Gross, 2015). The more people reappraise instead of suppressing, the better they face the daily events and the higher their opportunity to experience positive affect (Gross & John, 2003). The results achieved in this study suggest that the well-being practices were successful in improving emotion regulation abilities: suppression decreased from t0 to t8 and reappraisal increased from t8 to follow-up. Finally, the grateful disposition also increased from t0 to t8 and the effects were maintained in follow-up. This was a result expected mainly for the gratitude list group, which also extended to the practice of recalling other types of episode: self-affirming, achieved goals, or meaningful ones.

In fact, it is worth noting that all groups improved well-being. No matter the content, simple recalling, elaborating, and writing was beneficial. This emphasizes that the important underlying factor is not to focus on achieved goals, self-affirmed strength or gratitude episodes, or other aspects, but rather on ‘savoring’ these experiences, as largely demonstrated by Diener and Biswas-Diener (2011).

Regarding what leads the most to an increase in well-being and a decrease in ill-being, the content analysis showed that overall participants recalled and elaborated more on episodes of relationships and achievements suggesting that those lead largely to the beneficial effects. However, the observed differences among groups suggest that relationships played the major role for the gratitude list and the meaningful things groups, competence for the self-affirmation and goal setting groups. Interestingly, episodes of autonomy were reported less than the other types of episodes by all groups except control. This could be linked with the specific instructions given about affirming personal strengths or self-managed goals. It seems like the participants focused more on the successful experiences than on the process of self-directing the course of action. Future research could deepen the knowledge of the underlying factors, perhaps asking participants the reasons for choosing exactly those episodes.

### Practical Implications

The results suggest that the proposed practices increase well-being and curb ill-being. Participants felt more need satisfied, self-compassionate, grateful, and improved their ability to regulate emotions. This would suggest that these well-being practices whose effectiveness has been demonstrated during the COVID-19 pandemic outspread could be helpful and applied in any other potentially stressful situation, mainly when uncertainty, social isolation, and doubts about the future arise.

The effects were maintained or even increased one month after the end of the 7-week practice. From a practical point of view, an immediate suggestion is to disseminate as much as possible the practice of recalling, elaborating and writing relevant episodes among students. A close possibility is to extend it to workers, laypersons, younger students, maybe also clinical populations, as suggested by previous research: the gratitude list has been found beneficial also for adolescents (Froh et al., 2008, 2009), prisoners (Deng et al., 2018), teachers (Chan, 2010) and the elderly (Killen & Macaskill, 2015). Self-affirmation was also effective with students and adults (for a review, see Cohen & Sherman, 2014). Goal setting was successfully applied in the fields of sport (for a review, see Jeong et al., 2021), health (for a review, McEwan et al., 2016), education (Bruhn et al., 2016) and behavior change (Epton et al., 2017).

Furthermore, while most previous research considered a single kind of practice adopted repeatedly over time, the study by Behzadnia and FatahModares (2020) suggests that a mix of activities should also work. Future applications should hence include different activities on different days or a combination of activities. It is possible that forcing one to think solely about gratitude episodes or fulfilled goals episodes could have been perceived as a limitation, which could have reduced the effects: a variation in the content to recall and elaborate could even increase the effectiveness.

### Limitations and Future Avenues

First, this study did not include a control group that did nothing (only filling out questionnaires) or did alternative activities or focused on negative episodes. Future research should consider a control group that is not involved in recalling positive episodes. For instance, as in Emmons and McCullough (2003), participants in the control group could be asked to list burdens and reflect on them. This would consent to compare more clearly the effects of thinking about positive over negative episodes, thus avoiding the confounding due to reporting a mix of episodes or even neutral ones. In addition, a passive control group (only asked to complete questionnaires) could also be included, with the aim of assessing the advantages/disadvantages of reflecting on positive/negative episodes compared to not doing ‘recall and elaborate’ activities. Second, the effects were found on well-being aspects, while it is unknown whether some performance indices should also be affected, for example, marks or scores in a standardized test of logical reasoning or attention, for example. Following the broaden-and-built theory (Fredrickson & Joiner, 2002), it is possible to speculate that the greater positivity will reflect in improved performances. Third, the participants were only university students attending psychology courses. Future research could test the effectiveness of these practices with different samples, including clinical populations. Forth, all variables were self-reported. Maybe in the future some objective assessment could be included, for instance, the breadth of thought–action repertoire, that is, asking the participants to list as many projects they have in the upcoming week (Fredrickson & Branigan, 2005). Fifth, in this study, participants were asked to do the same practice over and over, as suggested by Emmons and McCullough (2003) and Froth et al. (2008), while in other research every week there was a different request (see, e.g., Behzadnia & FatahModares, 2020). Future studies could verify whether repetitive tasks were perceived as boring and introduce a mix of practices, including gratitude list, goal setting, and self-affirmation, in different weeks to verify if the effects could be even greater. Six, some moderating variables, not assessed in this study, should have affected the results. Among them are the religious beliefs, that should have prompted toward expressing gratitude, achieving goals, developing personal strengths. Future research should include this measure and assess its moderating role.

## Conclusions

Recalling, elaborating, and writing weekly on recent personal and potentially enriching episodes was beneficial: it increased well-being and decreased ill-being. This complements the bulk of the literature showing that a supportive social environment improves need satisfaction (see, e.g., Reeve, 2009, 2016; Ryan & Deci, 2017) and that trainings could raise self-compassionate attitudes and emotion regulation (see, e.g., Barnard & Curry, 2011; Emmons & McCullough, 2003; Horn et al., 2011). Alternatively, or complementing these interventions, something can be done personally to increase the experience of need satisfaction, foster self-compassion, improve emotion regulation abilities, and maybe also take greater advantage of need supportive environments. The practice of recalling, elaborating, and writing weekly episodes seems to be a promising way.

## Appendix

The final questions to assess maintenance and percentages.

- Did you perform the well-being practices again after the 7-weeks?
- No, I never thought about them 26%
- I thought about them, but I did not do them 41%.
- I did them one or two times, 18%
- I did them many times, 11%
- I did them always and I will continue 4%
- Did you suggest friends or family members to do this kind of practice?
- No 58%
- One person 27%
- More people 13%
- Many people
- Many people and I think I will continue to suggest 2%
- How satisfied are you with these practices?
- Not at all 4%
- A little 23%
- Enough 42%
- Much 27%
- Very much 4%
